# Oxyphenbutazone improves memory and learning impairments in LPS-induced neurotoxicity via modulating TGF-β/NF-κB/IκB-α pathways: In silico and in vivo study

**DOI:** 10.1371/journal.pone.0337611

**Published:** 2026-01-20

**Authors:** Khalid Saad Alharbi, Muhammad Afzal, Sattam Khulaif Alenezi, Reem ALQahtani, Tariq G. Alsahli, Faisal Imam, Imran Kazmi

**Affiliations:** 1 Department of Pharmacology and Toxicology, College of Pharmacy, Qassim University, Buraydah, Al Qassim 51452, Saudi Arabia; 2 Department of Pharmaceutical Sciences, Pharmacy Program, Batterjee Medical College, P.O. Box 6231, Jeddah 21442, Saudi Arabia; 3 Department of Pharmaceutical Sciences, College of Pharmacy, Princess Nourah Bint Abdul Rahman University, Riyadh, KSA; 4 Department of Pharmacology, College of Pharmacy, Jouf University, Sakaka, Aljouf, 72341, Saudi Arabia; 5 Department of Pharmacology and Toxicology, College of Pharmacy, King Saud University, Riyadh, 11452, P.O. Box 145111, Saudi Arabia; 6 Department of Biochemistry, Faculty of Science, King Abdulaziz University, Jeddah, 21589, Saudi Arabia; Universite Cote d'Azur, FRANCE

## Abstract

**Objective:**

Neuroinflammation and oxidative stress play key roles in cognitive decline and memory impairments. This study employed a lipopolysaccharide (LPS)-induced neurotoxicity model and in silico methods, including in silico docking and dynamic simulations, to assess the therapeutic potential of Oxyphenbutazone (OPB).

**Materials and methods:**

Wistar rats were categorized as control, LPS-only section, LPS + OPB receiving low (35 mg/kg) and high (70 mg/kg) doses of OPB, and only OPB (70 mg/kg) doses. Behavioral assessments (Y-maze and Morris water tests) were used to assess cognitive ability. The levels of neuroinflammatory markers [Interleukin-6 (IL-6), IL-1β, and tumor necrosis factor-alpha (TNF-α)] and oxidative stress modulators [Superoxide dismutase (SOD), glutathione (GSH), catalase (CAT), and malondialdehyde (MDA)] were estimated. Additionally, transforming growth factor-beta (TGF-β), nuclear factor-kappa B (NF-κB), and inhibitor of kappa B-alpha (IκB-α) levels were evaluated. In silico analyses, such as molecular docking and dynamic simulations, were used to evaluate the stability of OPB and target molecules.

**Results:**

Cognitive performance improved after OPB treatment, and the levels of proinflammatory cytokines, antioxidants, TGF-β, NF-κB, and IκB-α were restored. Additionally, in silico analyses illustrated favorable and stable interactions between OPB and the target molecules NF-κB and IκB-α.

**Conclusion:**

These findings suggest the therapeutic potential of OPB in mitigating neurotoxicity and the associated cognitive disabilities.

## Introduction

Neurodegenerative ailments are characterized by neuronal loss, which results in disturbances in brain function [1–4]. Neurodegenerative diseases, mainly Alzheimer’s disease (AD), Parkinson’s disease (PD), Huntington’s disease, and amyotrophic lateral sclerosis (ALS), are significant global health burdens owing to their impact on millions of people. This burden is substantial and is expected to increase with an ageing global population. These clinical conditions lead to profound disabilities and are a prominent cause of death worldwide, affecting approximately 15% of the global population [5,6]. Neurotoxicity is caused by events such as neuroinflammation, triggered by the activation of microglia, astrocytes, and reactive oxygen species, causing the blood-brain barrier and subsequent neurotoxicity [7].

Neurotoxicity leads to significant memory impairment and cognitive deficits caused by multifaceted mechanisms such as oxidative stress and inflammation [8]. Memory and learning impairments progress with increasing age and have a notable effect on the routine lives of patients [9]. Neuroinflammation is a key factor that contributes to the development of neurotoxicity. During this process, microglia and astrocytes are triggered, leading to the release of inflammatory modulators that cause neuronal injury [10,11]. Lipopolysaccharide (LPS)-induced neurotoxicity has been widely used to study neurological disorders. Neuroinflammation and dopaminergic degeneration are key factors contributing to neurodegeneration in rodents [12]. Additionally, LPS administration leads to cognitive impairment and memory deficits in animals, making it favorable for evaluating behavioral parameters of neurological complications. It mimics mechanisms, such as the activation of inflammatory cytokines and oxidative species formation, which are key events in the pathophysiology of neurotoxicity and associated cognitive deficits [13,14].

Oxyphenbutazone (OPB) is a metabolite of phenylbutazone that selectively inhibits cyclooxygenase (COX) enzymes [15]. OPB is an effective anti-inflammatory drug with both analgesic and antipyretic properties [16]. OPB was reported to mitigate cardiotoxicity in a murine model by eliminating oxidative stress and suppressing the nuclear factor-kappa B (NF-κB)/inhibitor of kappa B-alpha (IκB-α) pathway [17]. Cognitive impairment is a major challenge under various neurological and clinical conditions. Moreover, current therapeutic approaches have limitations and are less efficient in managing cognitive dysfunction [18,19].

Thus, identification of novel therapeutic agents is essential for the management of neurodegenerative disorders. Although OPB is known for its anti-inflammatory effects, its neuroprotective properties have not yet been explored in neurodegenerative diseases. This study aimed to assess the neuroprotective role of OPB in preventing the memory and learning deficits associated with LPS-induced neurotoxicity. This study aimed to examine the modulatory role of OPB on the transforming growth factor-beta (TGF-α)/NF-κB/IκB-α protein signalling pathway through cumulative behavioral, biochemical, and oxidative stress studies in rats, molecular docking, and dynamic simulations to confirm target interactions in silico.

## Materials and methods

### Animals

Wistar rats were housed in different cages at optimum temperature (22–25°C), humidity (45–55%), and light: dark cycle (12:12h). Open access to animal food pellets was provided ad libitum during the trial. The protocol was performed according to the guidelines of the Institutional Ethics Committee and the ARRIVE guidelines for research [20]. This study was conducted in strict accordance with the recommendations outlined in the Guide for the Care and Use of Laboratory Animals of the National Committee of Bioethics and Batterjee Medical College, Jeddah, Saudi Arabia. The research protocol was approved by the Institutional Research Board (IRB) of Batterjee Medical College, Jeddah, Saudi Arabia, with research proposal code (RES-2024–0069).

### Inclusion and exclusion criteria

Healthy Wistar rats (150–200 g) were included in this study, and animals with any symptoms of illness were excluded prior to group allocation. Rats that did not meet the established inclusion criteria were excluded from the study. Additionally, in the in silico experiments, only X-ray crystallography-resolved proteins with < 3.0 A° resolution were included, excluding the co-crystallized ligands.

### Chemicals

OPB and LPS were obtained from Sigma Ahldrich USA. Cytokines, including Interleukin-6 (IL-6), IL-1β, and tumor necrosis factor-alpha (TNF-α), TGF-β, NF-κB, and IκB-α, were detected using a rat enzyme-linked immunosorbent assay (ELISA) kit sourced from MyBioSource, Inc. USA.

### Acute toxicological study

Acute toxicity evaluation of OPB was conducted according to the guidelines outlined in the OECD ANNEX-423 protocol, which is a standard procedure for examining the safety profile of drugs. In this study, OPB was administered orally at doses of 35 and 70 mg/kg, in accordance with a previously published safe dose, to Wistar rats to evaluate the acute toxicity of these doses. Moreover, pharmacokinetic properties, including absorption, distribution, metabolism, excretion, and toxicity (ADMET), of OPB were evaluated using pkCSM ADMET descriptors (Table 1). The findings of this study revealed that OPB administration to experimental rats did not result in any signs of toxicity or mortality, indicating the safety profile of the drug [21].

**Table 1:** Predicted ADMET properties of Oxyphenbutazone.

Property	Model Name	Predicted Value	Unit
**Absorption**	Water solubility	−4.212	Numeric (log mol/L)
	Caco2 permeability	1.355	Numeric (log Papp in 10^6^ cm/s)
	Intestinal absorption (human)	93.792	Numeric (% Absorbed)
	Skin Permeability	−2.776	Numeric (log Kp)
	P-glycoprotein substrate	No	Categorical (Yes/No)
	P-glycoprotein I inhibitor	Yes	Categorical (Yes/No)
	P-glycoprotein II inhibitor	No	Categorical (Yes/No)
**Distribution**	VDss (human)	−0.106	Numeric (log L/kg)
	Fraction unbound (human)	0	Numeric (Fu)
	BBB permeability	0.018	Numeric (log BB)
	CNS permeability	−2.236	Numeric (log PS)
**Metabolism**	CYP2D6 substrate	No	Categorical (Yes/No)
	CYP3A4 substrate	Yes	Categorical (Yes/No)
	CYP1A2 inhibitor	Yes	Categorical (Yes/No)
	CYP2C19 inhibitor	Yes	Categorical (Yes/No)
	CYP2C9 inhibitor	Yes	Categorical (Yes/No)
	CYP2D6 inhibitor	No	Categorical (Yes/No)
	CYP3A4 inhibitor	Yes	Categorical (Yes/No)
**Excretion**	Total Clearance	0.157	Numeric (log ml/min/kg)
	Renal OCT2 substrate	No	Categorical (Yes/No)
**Toxicity**	AMES toxicity	Yes	Categorical (Yes/No)
	Max. tolerated dose (human)	−0.096	Numeric (log mg/kg/day)
	hERG I inhibitor	No	Categorical (Yes/No)
	hERG II inhibitor	Yes	Categorical (Yes/No)
	Oral Rat Acute Toxicity (LD50)	2.241	Numeric (mol/kg)
	Oral Rat Chronic Toxicity (LOAEL)	1.735	Numeric (log mg/kg_bw/day)
	Hepatotoxicity	No	Categorical (Yes/No)
	Skin Sensitisation	No	Categorical (Yes/No)
	T.Pyriformis toxicity	0.552	Numeric (log ug/L)
	Minnow toxicity	0.734	Numeric (log mM)

ADMET, absorption, distribution, metabolism, excretion, and toxicity; BBB, blood-brain barrier; CNS, central nervous system; CYP, Cytochrome; hERG, human ether-go-go gene; LD50, lethal dose 50; OCT2, organic cation transporter 2; VDss, volume of distribution.

### Experimental

A total of 30 rats were randomized into five groups (n = 6).

- Group 1 (control) received a single intraperitoneal dose of normal saline (0.2 ml) for 7 days.
- Group 2 (LPS-injected group) received 1 mg/kg LPS (i.p.) for 7 days.
- Group 3 (LPS + OPB – Low dose) received 1 mg/kg LPS (i.p.), followed by 35 mg/kg OPB orally for the subsequent 7 days.
- Group 4 (LPS + OPB – High dose) received 1 mg/kg LPS (i.p.), followed by 70 mg/kg OPB orally for the subsequent 7 days.
- Group 5 (OPB – High dose) received only 70 mg/kg of OPB orally for the subsequent 7 days.

**Fig 1:**
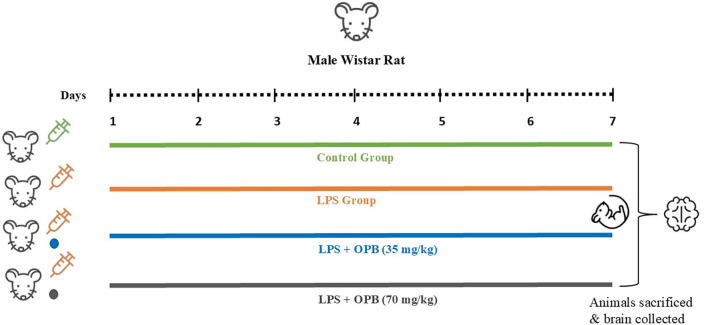
Experimental outline.

Following the experiment, the rats were anesthetized with intraperitoneal ketamine/xylazine (75/10 mg/kg, i.p.) [22]. All efforts were made to minimize suffering. The rats were sacrificed by cervical dislocation, and their brains (hippocampi) were preserved for subsequent biochemical analysis. This was followed by dissection of the hippocampus, which was stored at −80°C after freezing using dry ice. Phosphate-buffered saline (0.2 M) was used to homogenize the hippocampal tissues, followed by centrifugation at 13000 rpm for 25 min (4°C) to separate the supernatant, which was then preserved at −70°C (Fig 1).

### Behavioural assessments

#### Y-maze test.

The metallic Y-shaped maze, with three arms named ‘A,’ ‘B,’ and ‘C,’ had a width of 10 cm, a height of 25 cm, and a length of 35 cm extending from a central platform and positioned at an angle of 120°. During the experiment, the rats were stationed on arm ‘A’ and given 5 mins for exploration. The entry into the arm was marked when all four limbs of the animals were within the novel arm. Spontaneous alteration behavior (SAB) encompasses the recurrent patterns of entry sequences observed within the arms. The arms were wiped to avoid residual odors after each session, and the percentage of SAB was calculated using the mathematical expression [23].


$$\text{SAB}\%=\frac{\text{Number}\;\text{of}\;\text{alterations}}{\text{Total}\;\text{number}\;\text{of}\;\text{entries}\;-\;\text{2}}\times \text{100}$$

#### Morris water maze (MWM) test.

The MWM test assessed the learning and behavioral capacity of the experimental animals using a cylindrical tool with a diameter of 120 cm and a height of 40 cm, provided with water at a level of 30 cm (24 ± 1 °C). The apparatus was partitioned uniformly into four equal parts and a white plate (10 × 10 cm^2^) was positioned at the center of the designated quadrant, 1 cm below the water surface. The activities of the experimental participants were recorded using a digital camera between 4 and 7^th^ day of the experiment. The ceiling time was three minutes for each attempt. The animals were allowed to seek a base for 3 minutes and rest for 30 seconds. The hidden platform was removed on the last day of an expert probe trial in which the elapsed time in the quadrant designated by the subject was noted for 90 seconds.

### Biochemical assays

#### IL-1β, IL-6, TNF-α, TGF-β, NF-κB, and IκB-α estimation.

The Enzyme-Linked Immunosorbent Assay (ELISA) is a widely used laboratory method for quantifying the protein levels of IL-1β, IL-6, TNF-α, TGF-β, NF-κB, and IκB-α in rat samples. These ELISA kits were based on microwell plates and pre-coated antibodies (Merck Life Sciences Pvt. Ltd., India). The experiment was initiated by coating the plate with antibodies followed by sample addition. Subsequently, a primary antibody was added to bind the target protein. Next, the secondary antibody linked to the enzyme was added. The secondary antibody then binds to the target protein to form a sandwich complex. Subsequently, a substrate for the enzyme is introduced, which catalyses a reaction that results in a chromogen. Tris-based washing buffer was used to wash the microwell plates and remove undesired molecules. The intensity of the color in the samples was measured using an ELISA reader at a wavelength of 450 nm.

#### Oxidative markers.

Superoxide dismutase (SOD) was quantified using a method discussed earlier [24]. The assay mixture was composed of the specimen (0.01 ml), buffer solution (0.10 ml) made of sodium pyrophosphate (pH 8.2, 0.050 M), phenol methosulfate (0.01 ml), and nitro blue tetrazolium (300 µL). The reaction was performed by adding NADH. After 90 s, glacial acetic acid (100 µL) was added. The chromogen was then separated by centrifugation. The chromatic intensity was measured at 560 nm using n-butanol as a reference.

Glutathione (reduced form, GSH) levels were estimated in the hippocampal homogenates. Trichloroacetic acid (TCA) was combined with homogenate supernatant in equal proportions and centrifuged at 4°C for 10 min at 1000 × g. The supernatant was mixed with 1000μl (0.3 M) disodium hydrogen phosphate and 250μl of 0.001 M 5,5`-dithiobis (2-nitrobenzoic acid). Absorbance was measured at 412 nm [25].

The malondialdehyde (MDA) levels were estimated as described by Jangra et al. [25]. 50 microliters of hippocampal homogenate were mixed with an equal concentration of 8.1% sodium dodecyl sulfate (SDS). Subsequently, acetic acid (20%) and thiobarbituric acid (0.8%) were added in a 1:1 ratio to the mixer. The obtained contents were transferred to an Eppendorf tube, kept under heat (95°C), allowed to reach room temperature, and later processed by centrifugation at 10,000 rpm (10 min). The absorbance was measured at 532 nm and is presented as nmol/mg.

To estimate catalase levels, a blend of 100 μL hippocampal homogenate supernatant and 2000 μL phosphate buffer (pH 7.0, 50 mM) was prepared. Subsequently, 1000 μL of hydrogen peroxide (30 mM) was added to this mixer and expressed as U/mg [26].

### Molecular docking (MD)

NF-κB and IκB-α were considered as targets for the MD analysis, and their FASTA format sequences were sourced from the National Centre for Biotechnology Information, followed by a BLAST search against the Protein Data Bank (PDB) to obtain similar sequences, and the top five to ten sequences were selected. The 3D structures of NF-κB (PDB ID: 1SVC) and IκB-α (PDB ID: 1NFI) were obtained from the PDB database, and structural validation was performed. The validation details for these proteins are summarized in Table 2. Side chains were generated, and absent residues were inserted using Chimera v1.16. Biovia Discovery Studio (V21.1.0.20298) was used to eliminate non-standard residues and unnecessary side chains. Grid was generated using AutoDockTools, Chimera, and Maestro and the active sites of target proteins were recognized using the Computed Atlas of Surface Topography of Proteins server (Table 3), whereas the grid parameters were described (Table 4). The ligands were cleaned and maintained in 3D MOL2 format using MarvinSketch v21.13. The structures were modified into pdbqt format using AutoDock Tools 1.5.6 and ADFRsuit, respectively. MD was executed using AutoDock Vina 1.2.3. The Biovia Discovery Studio visualizer was used to visualize the protein-ligand complexes. Maestro 12.3 (academic edition) and LigPlus 1.2. were used to create 2D and 3D images (Table 5).

**Table 2:** Comparison of standard values with the retrieved protein for protein validation shortlisted for docking analysis.

Parameters	Details		Standards
**Target**	**NFKβ**	**IKβ-α**	**–**
Protein Id and	**1SVC**	**1NFI**	**–**
Method of experiment	X-RAY Diffraction	X-RAY Diffraction	X-RAY Diffraction
Mutation	No	No	No
Resolution	**2.60 Å**	2.70 **Å**	Near about 3.00 **Å**
wwPDB Validation	Better	Better	Better
Co-Crystal Ligand	Absent	Absent	–
Ramchandran Plot (by PROCHECK server) Residues in favoured + Allowed regions	90.0%	79.80	>88%

**Table 3:** Amino acid’s active cites.

Protein ID	The active sites amino acids
1SVC	LYS52, ARG54, GLY55, SER243, ALA248, SER249, ASN250, LEU251, ARG336, GLU341, THR342, SER343, GLU344.
1NFI	LYS286, GLN29, ARG30, GLY31, MET32, ARG33, PHE34, ARG35, TYR36, LYS37, CYS38, GLU39, ARG41, SER42, ALA43, GLY44, SER45, ILE46, PRO47, GLU49, ARG50, SER51, THR52, ASP53, THR54, THR55, LYS56, THR57, HIS58, VAL91, GLY92, ASN115, LEU116, GLY117, ILE118, GLN119, ASN186, LYS221, GLU222, ASP223, ILE224, GLU225, ARG236, GLY237, SER238, PHE239, GLN241, PRO275, SER276, ARG278, THR257, TRP258, GLY259, ARG260, PRO261, SER262, THR263, GLN266

**Table 4:** Grid parameter.

Sr.no	Protein Id	Centre Coordinates			Size Coordinates		
		x	y	z	x	y	z
1	1SVC	34.401	9.319	37.650	20	20	20
2	1NFI	2.813	47.983	18.942	30	30	30

**Table 5:** Docking Score and intermolecular interactions of ligands with NF-Kβ (PDB id 1SVC) and IKβ-α (PDB id 1NFI).

SR. NO.	NAME OF COMPOUND	BINDING ENERGY (kcal/mol)	TYPE OF INTERACTION	RESIDUE ID	DISTANCE (IN A0)
1	1SVC_OXYPHENBUTAZONE	−5.397	Hydrophobic Interactions	ARG54P	54P
				GLU341P	341P
			Hydrogen Bonds	LYS52P	52P
				THR342P	342P
				GLU344P	344P
				GLU344P	344P
			π-Cation Interactions	LYS52P	52P
2	1NFI_OXYPHENBUTAZONE	−6.668	Hydrophobic Interactions	LYS28A	28A
				ARG50A	50A
				ASP223A	223A
				TRP258F	258F

### Molecular dynamics simulation (MDS)

Desmond 2020.1 was employed to perform MDS for 1SVC (NFKβ) and 1NFI (IKβ-α) in the Apo state and with OPB represented as 1SVC_ OPB and 1NFI_ OPB, respectively [27–29]. TIP3P water molecules with a force field (OPLS-2005) and a solvent system (explicit) were used, and a boundary box was created [30]. The system was supplemented with Na+ and NaCl solutions. Initially, the system was equilibrated using the NVT ensemble (10 ns) followed by the NPT ensemble (12 ns). The NPT ensemble at different temperatures with a resting period (1.0 ps) and 1 bar of pressure was conFig.d using Nose–Hoover chain coupling [31]. The Martyna-Tuckerman-Klein chain coupling scheme was employed for pressure stabilization, with a resting duration of 2 PS [32]. Electrostatic interactions were determined by the particle mesh Ewald method, and the radius of the Coulomb interactions was set (9 Å) [32]. The final run was performed for 100 ns. The root-mean-square deviation (RMSD), radius of gyration (Rg), root-mean-square fluctuation (RMSF), and number of hydrogen bonds (H-bonds) were determined to evaluate the stability of the MDS. Binding free energy analyses were performed using a previously described method (Table 6) [33].

**Table 6:** Components of binding free energy for the 1SVC_OPB and 1NFI_OPB measured by MM-GBSA.

Energies (kcal/mol)	1SVC_Oxyphenbutazone	1NFI_Oxyphenbutazone
**ΔG**_**bind**_	−14.75 ± 3.22	−42.63 ± 2.33
**ΔG**_**bind**_ **Lipo**	81.57 ± 21.20	37.47 ± 12.72
**ΔG**_**bind**_ **vdW**	3.27 ± 3.30	1.79 ± 1.04
**ΔG**_**bind**_ **Coulomb**	−0.40 ± 0.26	−0.70 ± 0.14
**ΔG**_**bind**_ **H**_**bond**_	−5.98 ± 1.39	−12.35 ± 1.40
**ΔG**_**bind**_ **SolvGB**	−71.68 ± 21.38	−37.85 ± 12.72
**ΔG**_**bind**_ **Covalent**	−20.99 ± 4.54	−29.40 ± 2.69

### Statistical analysis

GraphPad Prism (version 8.0.2) was used for data analysis, with standard error of the mean (SEM). One-way analysis of variance (ANOVA) and Tukey’s post-hoc analysis were used for biochemical analyses. The results of the behavioral parameter (MWM) were processed using a two-way ANOVA, followed by the Bonferroni test. Statistical significance was set at P < 0.05.

## Results

### Acute toxicity study

The acute toxicity of OPB was examined in Wistar rats following the toxicological assessment. The experimental rats did not exhibit any signs of illness, toxicity, or mortality throughout the experiment after administration of 35 and 70 mg/kg of OPB. The monitoring period lacked any adverse effects, which supported the safety of the drugs at both concentrations. Based on these findings, 35 and 70 mg/kg OPB were selected for subsequent analysis.

### Behavioural tests

#### Y maze test.

**Fig 2:**
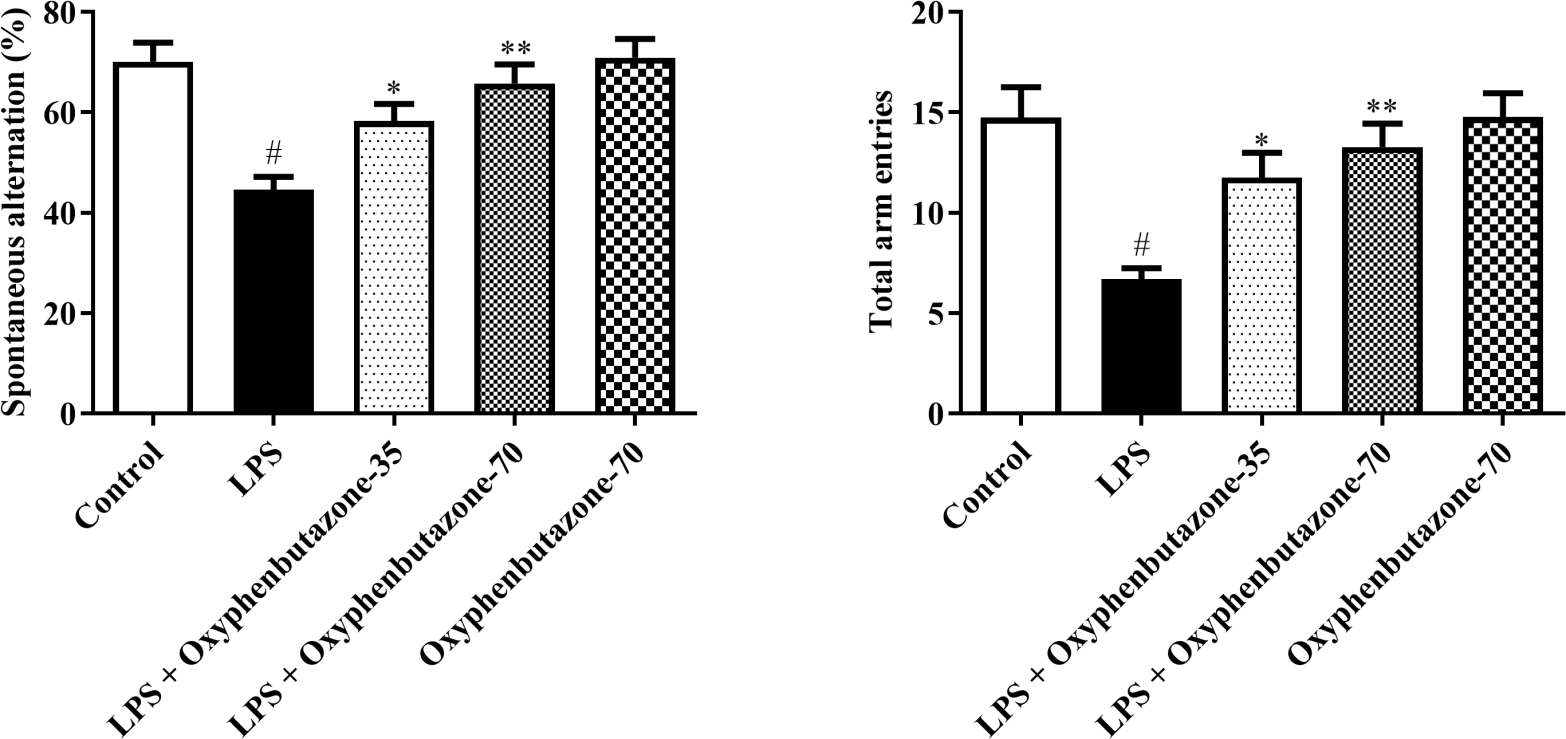
Impact of OPB on Y-maze-spontaneous alternation test and total arm entries in rats. In quantifying statistical data, this study reports mean ± S.E.M. (n = 6). A one-way ANOVA followed by Tukey’s post hoc analysis showed statistically significant findings compared to the LPS group: #P < 0.01 Vs control group; *P < 0.05, **P < 0.001.

SAB% and the total number of entries were markedly lower in rats treated with LPS than in the control group rats, indicating memory impairment due to LPS administration. In contrast, OPB at both doses significantly increased SAB% [F (4, 25) = 9.729; (P < 0.0001)] and the total number of entries [F (4, 25) = 8.106; (P = 0.0002)] when collated with LPS-treated rats. However, the OPB alone-treated group did not demonstrate any significant variations in SAB% and the total number of entries compared to the control group. These findings demonstrate that OPB exerts neuroprotective effects against LPS-induced memory impairment (Fig 2).

#### Morris water maze test.

**Fig 3:**
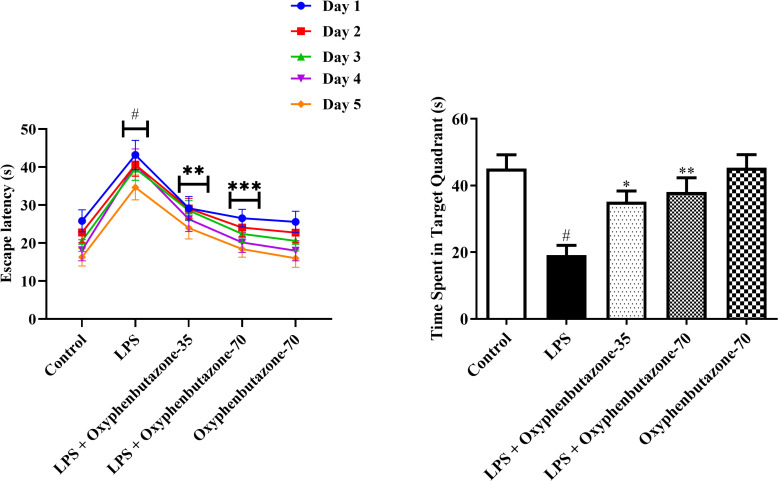
Impact of OPB on Morris water maze-escape latency and time spent in the target quadrant in rats. In quantifying statistical data, this study reports mean ± S.E.M. (n = 6). A two-way ANOVA for escape latency, along with a Bonferroni post hoc test for Morris water maze, and a one-way ANOVA followed by Tukey’s post hoc analysis for the duration spent in the target section, showed statistically significant results compared to the LPS group: #P < 0.01 Vs control group; *P < 0.05, **P < 0.001, ***P < 0.0001.

The MWM test was used to determine cognitive ability (Fig 3). During the experiment, LPS-administered rats showed significantly increased escape latency at all intervals compared with the normal group. However, OPB at both high and low concentrations markedly decreased the escape latency [F (4, 125) = 40.52; (P < 0.0001)] compared to LPS group. Moreover, OPB administration markedly increased the time spent in the target quadrant compared to LPS-treated rats [F (4, 25) = 8.174; (P = 0.0002)]. In contrast, the rats administered only OPB exhibited non-significant alterations in escape latency and time spent in the target quadrant compared to the control group.

### Biochemical assays

#### Assessment of oxidative markers.

**Fig 4:**
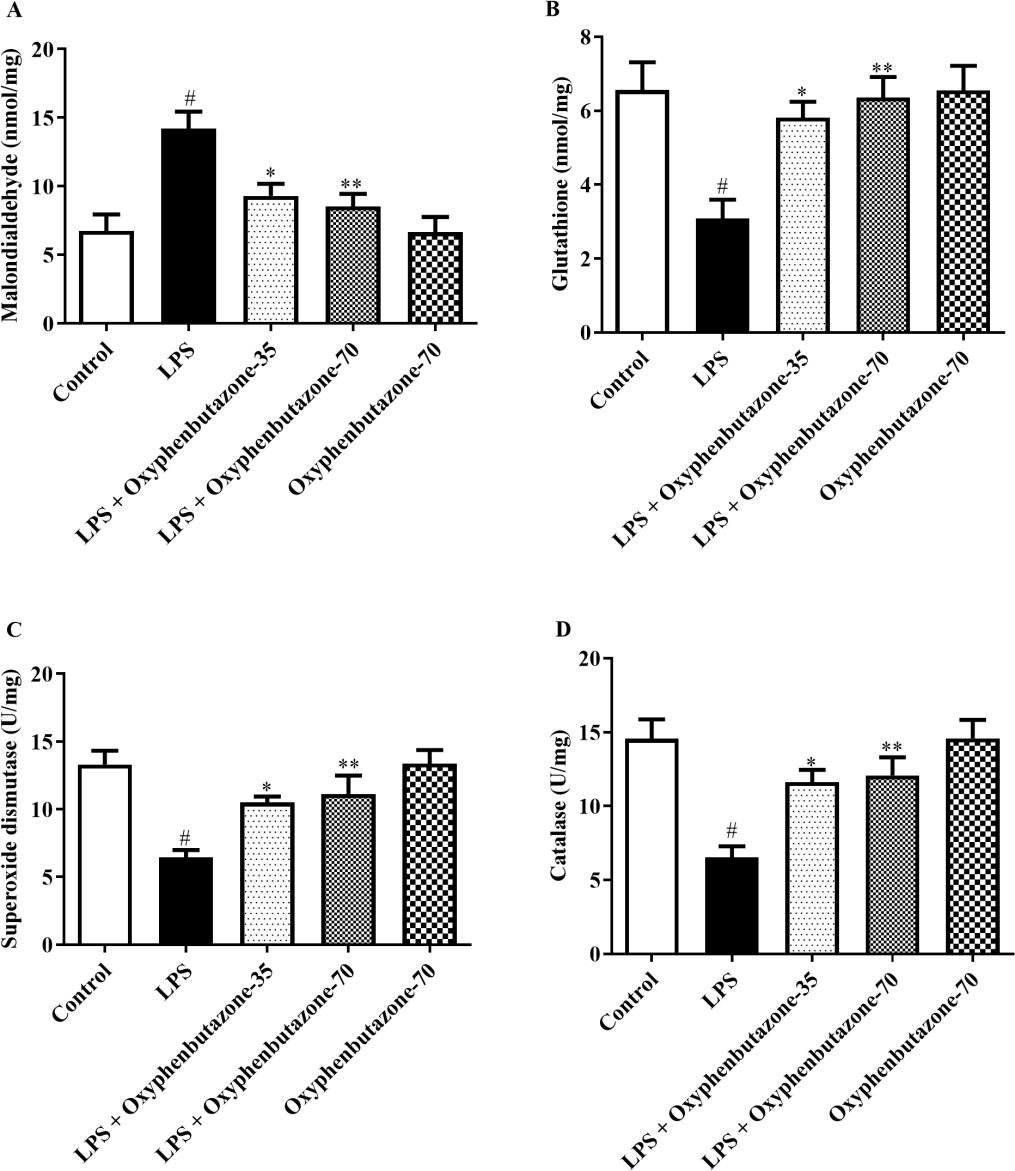
Impact of OPB on oxidative molecules (A) Malonaldehyde (MDA), (B) Glutathione (GSH), (C) Superoxide dismutase (SOD), and (D) Catalase (CAT) in rats. In quantifying statistical data, this study reports mean ± S.E.M. (n = 6). A one-way ANOVA followed by Tukey’s post hoc test revealed statistically significant results compared to the LPS group: #P < 0.01 Vs control group; *P < 0.05, **P < 0.001.

The assessment of antioxidant activity revealed notable changes in oxidative stress markers across all experimental groups, except for the control group. LPS administration notably reduced the levels of SOD, GSH, and CAT. However, MDA levels increased in LPS-treated rats, consistent with the control group, indicating increased oxidative stress (Fig 4A). Treatment with both low (35 mg/kg) and high (70 mg/kg) doses of OPB markedly elevated the levels of antioxidants, including SOD [F (4, 25) = 9.117; (P = 0.0001)], GSH [F (4, 25) = 6.205; (P = 0.0013)], and CAT [F (4, 25) = 8.993; (P = 0.0001)] in relation with only LPS-treated rats (Fig 4B–4D). However, MDA levels decreased after OPB treatment at both doses [F (3, 20) = 8.999; (P = 0.0006)]. Nevertheless, in comparison with the control group, the OPB-only experimental group did not experience any significant changes in SOD, GSH, CAT, or MDA levels.

#### Analysis of neuroinflammatory cytokines.

**Fig 5:**
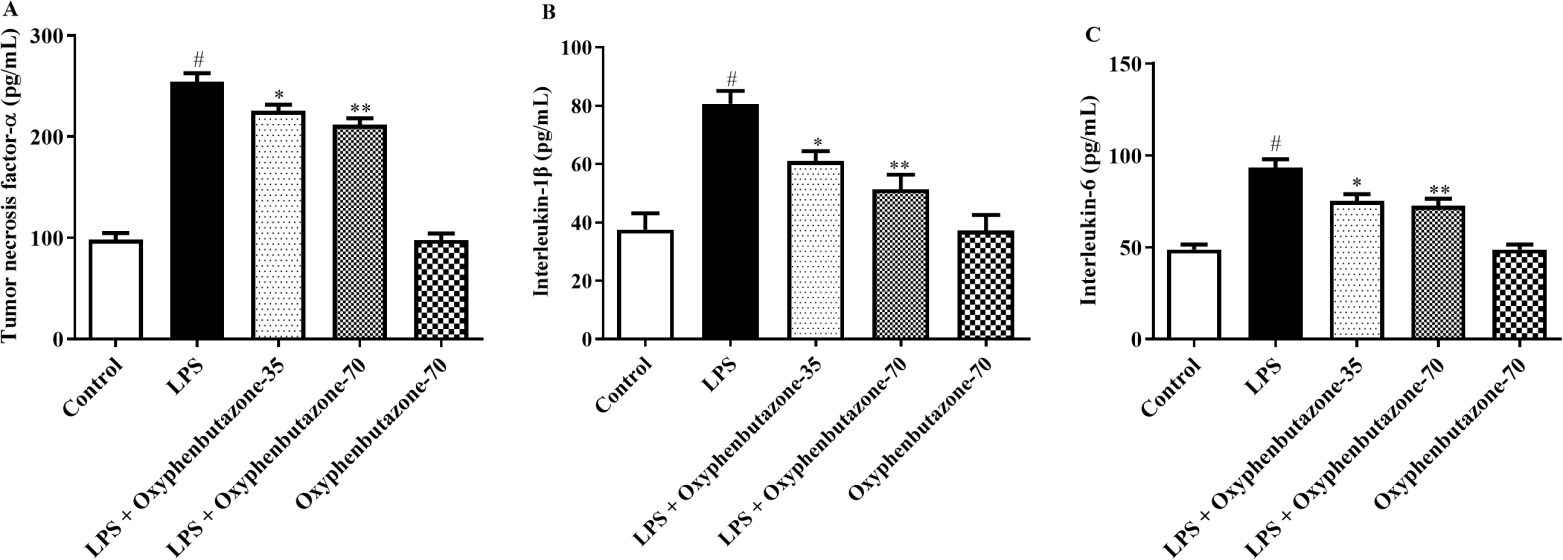
Impact of OPB on inflammatory modulators (A) Tumor necrosis factor-α (TNF-α), (B) interleukin-1β (IL-1β), (C) Interleukin-6 in rats. In quantifying statistical data, this study reports mean ± S.E.M. (n = 6). A one-way ANOVA followed by Tukey’s post hoc test showed statistically significant results compared to the LPS group: #P < 0.01 Vs the control group; *P < 0.05, **P < 0.001.

Analysis of neuromodulators revealed notable alterations in their levels after LPS administration. IL-1β, IL-6, and TNF-α levels were markedly increased in LPS-administered rats compared with those in the control group. This increase indicates an inflammatory response induced by LPS. Conversely, OPB treatment at both concentrations (35 and 70 mg/kg) strongly mitigated inflammatory responses in the LPS-administered group. However, the levels of IL-1β [F (4, 25) = 14.30; (P < 0.0001)], IL-6 [F (4, 25) = 27.58; (P < 0.0001)], and TNF-α [F (4, 25) = 119.4; (P < 0.0001)] markedly decreased after OPB treatment compared to the LPS-only group (Fig 5A–5C). Conversely, non-significant variations in the levels of these cytokines were observed in the rats treated with OPB only compared with the control group.

### Evaluation of NF-κB/IκB-α

**Fig 6:**
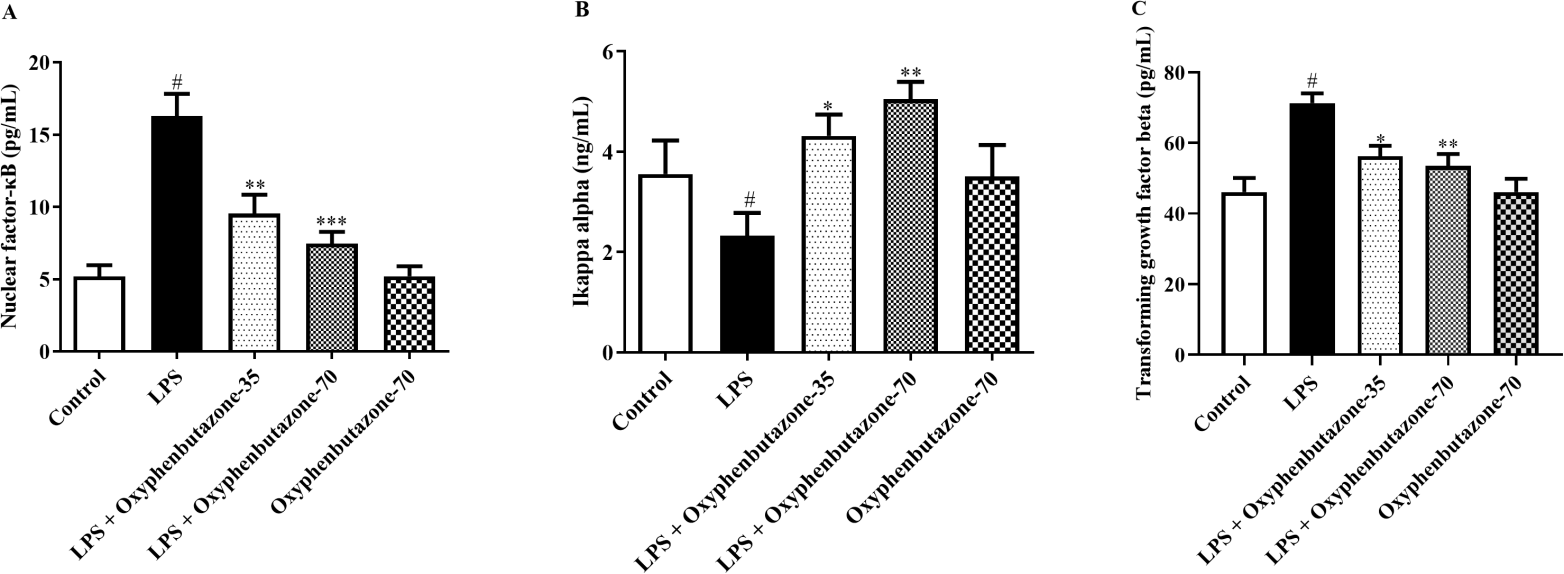
Impact of OPB on inflammatory markers (A) Nuclear factor-kappa B (NF-κB), (B) I kappa B alpha (IκB-α), and (C) Transforming growth factor beta (TGF-β) in rats. In quantifying statistical data, this study reports mean ± S.E.M. (n = 6). A one-way ANOVA followed by Tukey’s post hoc test showed statistically significant results compared to the LPS group: #P < 0.01 Vs control group; *P < 0.05, **P < 0.001, ***P < 0.0001.

The evaluation of NF-κB and IκB-α levels defined the pro-inflammatory response triggered by LPS administration (Fig 6A and 6B). The rats treated with LPS exhibited a marked elevation in NF-κB and degradation of IκB-α levels compared to the control group rats. Conversely, OPB intervention at low and high dose concentrations (35 and 70 mg/kg) decreased the levels of NF-κB [F (4, 25) = 17.87; (P < 0.0001)] and restored the level of IκB-α [F (4, 25) = 3.818; (P = 0.0148)] compared to the LPS-only group. In contrast, the concentrations of NF-κB and IκB-α were not markedly altered in the OPB alone group compared with those in the control group.

### Examination of the TGF-β signalling pathway

The LPS-treated group demonstrated a notable elevation in TGF-β expression compared to that in the control group, indicating activation of the anti-inflammatory pathway in response to inflammation triggered by LPS. During the experiment, TGF-β expression levels significantly decreased after OPB treatment at both high and low doses (35 and 70 mg/kg) compared with those in the LPS-only group [F (3, 20) = 10.06; (P = 0.0003)] (Fig 6C). Conversely, the expression levels of TGF-β did not show any significant changes in the OPB alone group compared to the control group.

### MD

**Fig 7:**
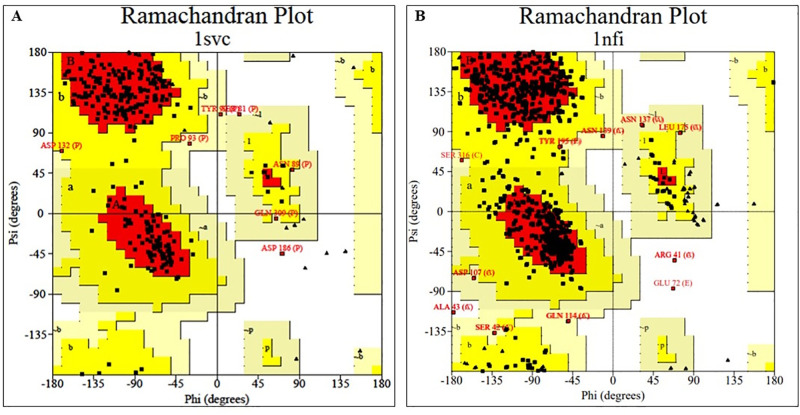
Ramachandran Plot (A) 1SVC and (B)1NRF sourced from the PROCHECK server.

**Fig 8:**
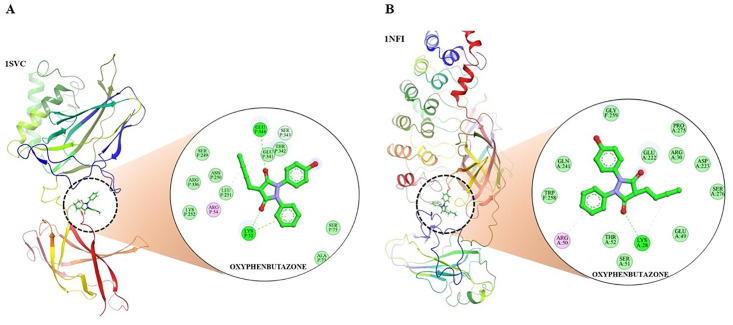
Docked conformation of ligand OPB with (A) 1SVC and (B)1NRF sourced from Maestro V12.8. and Biovia Discovery Studio.

MD was used to examine the binding compatibility of the ligands and their interactions with key residues in the binding region of the protein (Fig 7A and 7B). OPB exhibited a binding energy of −5.397 kcal/mol (Table 4) when interacting with NF-κB (PDB ID: 1SVC). It forms hydrophobic interactions with ARG54 and GLU341 while establishing hydrogen bonds with LYS52, THR342, and GLU344. In addition, π-cation interactions have been observed with LYS52. In contrast, OPB demonstrated a relatively strong binding energy of −6.668 kcal/mol with IκB-α (PDB ID: 1NFI). Hydrophobic interactions were observed with LYS28A, ARG50A, and ASP223A, whereas TRP258F exhibited hydrogen bonding. Molecular docking results indicated advantageous binding affinities and significant interactions between the ligand and target proteins, offering valuable insights into the inhibitory effects of OPB against LPS-induced neurotoxicity (Fig 8A and 8B).

### MDS

**Fig 9:**
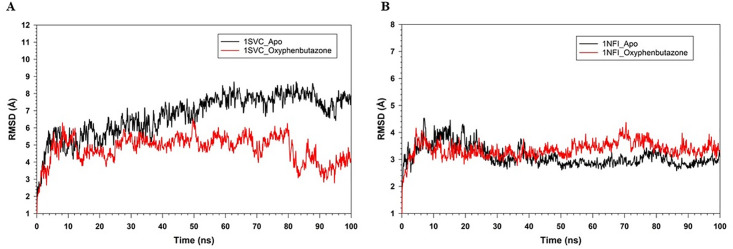
MD simulation of Cα backbone for 100 ns trajectories (A) 1SVC_Apo (Black) and 1SVC_OPB (red), (B) 1NFI_Apo (Black) and 1NFI_OPB (red).

The Desmond software suite was used to perform MDS for 100 ns at 300 K to assess the stability of the protein-ligand complexes generated by docking of OPB with 1SVC and 1NFI proteins. The root mean square deviation (RMSD) of the Cα-backbone atoms for both 1SVC (PDB ID: 1SVC) and 1NFI (PDB ID: 1NFI) (Fig 9A) indicates the stability for both proteins, with mean 1SVC and 1NFI with average RMSD values of 2.35 Å and 3.10 Å, respectively. RMSD of OPB within the active sites of 1SVC (1.75 Å) and 1NFI (4.02 Å) (Fig 9B). With respect to 1NFI, a ligand shift from its initial binding position to a new binding mode was observed during the first 15 ns. However, throughout the simulation, the ligand remained stable in the new binding orientation after the initial shift.

**Fig 10:**
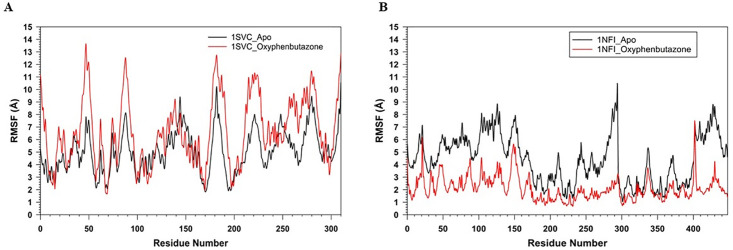
MD simulation of RMSF of Cα backbone for 100 ns trajectories A) 1SVC_Apo (Black) and 1SVC_OPB (red) (B) 1NFI_Apo (Black) and 1NFI_OPB (red).

**Fig 11:**
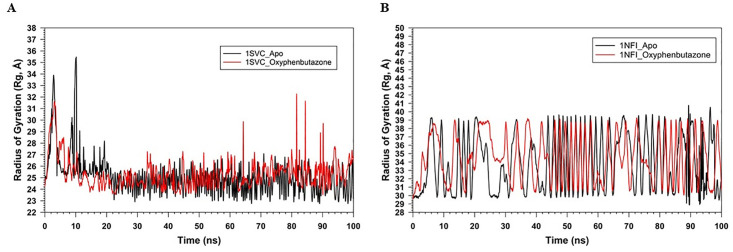
MD simulation of radius of gyration (Rg) of Cα backbone analysis for 100 ns trajectories (A) 1SVC_Apo (Black) and 1SVC_OPB (red), (B) 1NFI_Apo (Black) and 1NFI_OPB (red).

**Fig 12:**
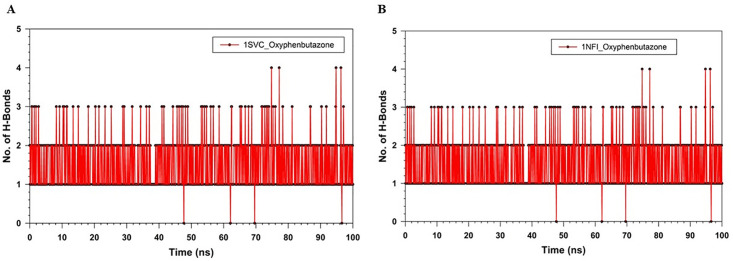
MD simulation analysis of hydrogen bond formation for 100 ns trajectories of (A) 1SVC_OPB (red), (B) 1NFI_OPB (red).

The plots of RMSF against the residue number (0–300) for 1SVC_Apo and 1SVC_OPB, ranging from 0 to > 15 Å, show how residue flexibility varies across the protein (Fig 10A). Both lines showed fluctuations along the number of residues, indicating variability in the flexibility of different parts of the protein. The peaks indicate flexible regions, whereas the troughs indicate rigidity. OPB binding alters the flexibility of specific residues, with some residues becoming more flexible and others more rigid. Notably, residues Lys77, Tyr90, Lys105, Arg187, Asn291, and Lys326 showed high RMSF, but were not in the catalytic site, which remained stable, rigid, and crucial for maintaining proper catalytic function. It has been found that no amino acids are present in catalytic sites with high RMSF values. These changes in flexibility may be key to protein function and may inform drug design strategies. Rigidity of the active site can be an intrinsic property of the protein, which is necessary for its specific function to ensure the precise positioning of catalytic residues and substrates. This suggests that proteins have a very specific and possibly narrow substrate range because flexibility often accommodates various substrates. The interaction of OPB with a protein induces changes in the flexibility of certain residues, which may be critical for the function or regulation of the protein. Understanding these changes is important for drug design and understanding the mechanistic basis of protein-ligand interactions. The RMSF of 1NFI_Apo and 1NFI_ OPB against residue number showed protein flexibility (Fig 10B). A higher RMSF score reflects flexible regions, often loops, or unstructured areas, whereas lower scores reflect more rigid, structured regions, such as alpha-helices or beta-sheets. OPB binding generally reduces protein flexibility, as indicated by lower RMSF values. Notable differences occurred around residues 100 and 200 and a sharp peak near 350, suggesting that these regions are affected by ligand binding, possibly due to allosteric effects. The sharp peak in the apo form near residue 350 may indicate an intrinsically disordered region that stabilizes upon binding, highlighting the key sites for interaction or modulation. This suggests that OPB binding stabilizes the protein structure, thereby reducing the flexibility of certain residues. The gyration (Rg) radii of 1SVC_Apo and 1SVC_OPB were over 100 ns, indicating protein compactness (Fig 11A). The Apo state is more stable with fewer fluctuations, whereas the OPB-bound form shows more variability, with sharp spikes suggesting moments of expanded conformation. The higher Rg values for the OPB-bound protein suggest that it adopts a less compact structure than that of the Apo form. This indicated that OPB binding induces conformational changes that generally lead to a more open protein structure with transient states of reduced compactness. The general trend of the Apo state is that it has fewer and lower peaks, suggesting that the unbound state of the protein is more stable, or that it experiences fewer conformational changes over the same time period. These data suggest that the binding of OPB to 1SVC induces conformational changes that are not present in the unbound state. These changes can be transient and lead to a less compact protein structure. The radius of gyration (Rg) over time for 1NFI in its apo form bound to OPB (Fig 11B). The Apo form exhibited significant fluctuations, indicating a flexible and variable structure. In contrast, the OPB-bound form showed a more stable Rg, suggesting that ligand binding imparted structural rigidity. The average Rg for the Apo form is 36.5 Å (±2.1 Å), while the OPB-bound form has a higher and more consistent Rg of 38.7 Å (±1.3 Å). The decreased variance in Rg values for the 1NFI_OPB complex supports the hypothesis that ligand binding stabilizes the specific conformational states of the protein. The observed fluctuations in Rg for 1NFI_Apo aligned with the anticipated dynamic nature of apo proteins, which often explore a wider conformational space in the absence of ligand binding. Our findings suggest that OPB binding exerts a stabilizing effect on the structure of 1NFI, potentially influencing its biological activity and ligand affinity through conformational selection. Number of hydrogen bonds over time for the 1SVC_OPB complex (Fig 12A). The graph fluctuates between 0 and 5 hydrogen bonds over 100 ns, typically stabilizing between 2 and 4 bonds. This fluctuation suggests a dynamic interaction between the protein and OPB, with hydrogen bond formation and breaking occurring throughout the simulation. The drop to zero hydrogen bonds could indicate moments when the drug partially or fully detached from the protein, suggesting a flexible binding site or multiple binding conformations. The number of H-bonds in the 1NFI_OPB complex was greater than 100 ns (Fig 12B). The number of H-bonds varied between zero and five, with the majority of the values falling between one and three. This fluctuation suggests that hydrogen bonds were consistently formed and broken throughout the simulation. The fact that the number of hydrogen bonds did not drop to zero suggests that at least one hydrogen bond was maintained throughout the simulation. Peaks reaching four or five bonds may correspond to transient molecular conformations, allowing more bonding interactions and providing insight into the stability and behavior of the complex.

## Discussion

This study demonstrated the neuroprotective potential of OPB against LPS-induced neurotoxicity in rats model. Both in vivo and in silico models have evaluated the effectiveness of OPB in mitigating neuroinflammation, reducing oxidative stress, and ameliorating cognitive deficits including learning and memory deficits.

According to the pkCSM ADMET prediction platform employed for ADMET prediction, OPB shows marginal intestinal absorption, which was approximately 93.79%, and also demonstrated minimal potential to cross the blood-brain barrier with a penalty of <1 [34]. Neurotoxicity study models developed by LPS administration are widely used to simulate the neuroinflammation pathways involved in the pathophysiology of various neurodisorders such as AD and PD. Upon administration, LPS activates microglia and astrocytes, which trigger pro-inflammatory modulators (IL-1β, IL-6, and TNF-α), causing the progression of neurological complications such as cognitive impairment [35,36]. The reproducibility and ability of the model to assess various compounds make it valuable for evaluating therapeutic products targeting inflammation-mediated neurological disorders. The antioxidative properties of OPB have been previously studied; however, its neuroprotective effects in cognitive impairment need to be evaluated [15,17].

Y-maze behavioral assessment is commonly employed to estimate spatial working memory and exploratory behavior. Previous studies have reported that LPS administration in rodents causes a decline in exploratory behavior in the Y-maze test, which is a sign of impaired cognitive function [37,38]. However, the current results demonstrated that treatment with OPB at both concentrations considerably mitigated LPS-induced behavioral impairment in the Y-maze test. LPS-induced neurotoxicity has been reported to impair cognitive function and memory, as illustrated by deficient activity in the MWM test in several studies [39–41]. OPB significantly reduced the neurotoxic effects of LPS in a rat model, as indicated by the better performance in behavioral assessments using MWM tests. The improvement in spatial memory and learning after OPB administration indicated that OPB may have a reversible effect on LPS-induced cognitive deficits.

LPS administration releases pro-inflammatory cytokines such as IL-6, which is one of the key mediators of neurodegenerative conditions [13,42]. The increase in IL-6 triggers neuroinflammation by stimulating microglia and astrocytes, causing the release of more cytokines and neurotoxic molecules [43]. Several studies have shown that suppressing the production of IL-6 results in neuroinflammatory decline, attenuation of behavioral dysfunction, and cognitive impairment [44–46]. LPS induces neurotoxicity through multifaceted interactions between various inflammatory mediators, with IL-1β and TNF-α playing a pivotal role. An increase in the levels of these inflammatory modulators causes neuroinflammation, whereas a reduction in the serum levels of IL-1β and TNF-α reverses neuroinflammation in rodents [45,47,48]. Similarly, this study showed a notable decline in IL-6, IL-1β, and TNF-α levels upon OPB administration at both high and low doses, indicating the potential of OPB to mitigate neuroinflammation associated with learning ability and cognitive decline.

Studies have illustrated that LPS administration alters the levels of antioxidants (GSH, SOD, and CAT) and MDA in the brain. LPS reduces the concentration of antioxidants but elevates MDA levels. These alterations lead to an increase in the levels of proinflammatory cytokines. Modulation of these antioxidants has been reported to alleviate oxidative stress-mediated neurotoxicity [49–51]. The current observations are in line with those of earlier studies, as LPS alters the normal levels of antioxidants; however, OPB at both concentrations significantly normalized the levels of these oxidative markers. These findings suggest that OPB may be an alternative treatment for mitigating neurotoxicity and the associated behavioral abnormalities. The TGF-β1 signalling pathway plays a vital role in neurotoxicity by activating Smad proteins and forming a complex that translocates to the nucleus to regulate gene expression [52]. Disturbances in TGF-β1 signalling lead to an increase in amyloid-beta deposition and neurodegeneration. In neurodegenerative disorders, the levels of TGF-β type II receptor are reduced in the brain, leading to neuronal injury due to the inhibition of TGF-β1/SMAD signalling [53]. The current findings show a sharp decline in TGF-β levels following LPS administration. On the other hand, normalization of TGF-β1 levels resulted in the reversal of neuronal damage, which aligns with our results where OPB significantly normalized TGF-β1 levels, showing its potential in mitigating neurotoxicity [54].

Several studies have shown that LPS administration triggers the NF-κB/IκB-α pathway, which facilitates the secretion of proinflammatory cytokines and subsequent neuronal impairment [13,55]. However, some therapeutic compounds have been reported to reduce neurotoxicity by inhibiting microglial activation and neuroinflammation and by suppressing the TLR4/NF-κB pathway [13,40]. In this study, LPS administration increased the NF-κB/IκB-α levels. However, treatment with OPB significantly reduced the concentration of NF-κB/IκB-α, indicating its therapeutic potential for ameliorating neurotoxicity.

A previous MD study showed that the inhibition of NF-κB-DNA binding has significant potential for treating neuroinflammation and associated neurotoxicity. Thus, molecular docking targeting NF-κB and IκB-α could be a promising strategy for developing novel therapeutics against neurotoxicity [56]. Additionally, Wang et al. (2018) [57] screened inhibitors of IKK-β using molecular docking, which could also be replicated to detect compounds that effectively bind to and stabilize IκB-α, leading to the discovery of novel pathways by targeting the NF-κB/IκB-α pathway. The present MD results illustrate that OPB has a strong binding affinity for NF-κB/IκB-α. Thus, in silico studies complement the findings of the in vivo model, emphasizing the potential of OPB in inhibiting neurotoxicity.

The MDS results strengthened the results of molecular docking, which showed favorable binding of OPB with the target proteins, NF-κB and IκB-α. During MDS, OPB formed stable complexes with the target molecules, 1SVC (NFKβ) and 1NFI (IKβ-α). However, the OPB-1SVC complex was comparatively more stable than OPB-The 1NFI, with persistent hydrogen bonds between residues (Ser208, Tyr254, and Lys326).

**Fig 13:**
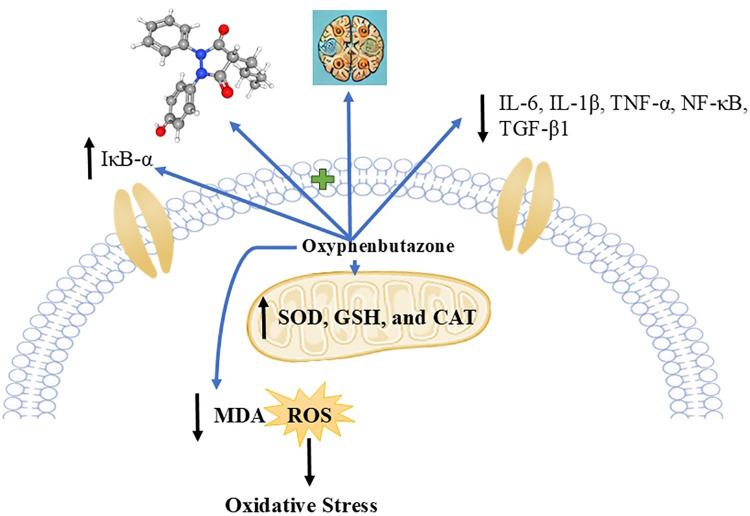
Hypothetical mechanism of OPB.

Through computational studies, crucial information was obtained on how exactly OPB exerts the neuroprotective properties of OPB against LPS-induced neurotoxicity. Several studies employing computational docking have revealed that OPB complexes of both NF-κB and IκB-α polypeptides are optimal and stable complexes that are vital for the inhibition of the inflammatory cascade. The binding energies between PB and NF-κB and IκB-α were found to be −5.397 kcal/mol and −6.668 kcal/mol by forming essential hydrogen bonds and hydrophobic interactions at their active sites. These interactions render the possibility of direct interference of OPB with NF-κB activation by stabilizing IκB-α and preventing its breakdown to inhibit the nuclear translocation of NF-κB and the subsequent transcription of pro-inflammatory cytokines. MDS further proved the stability of these protein-ligand complexes throughout the 100-ns simulation period with continued hydrogen bonding, reduction of the flexibility of critical areas, and lower RMSD values, which is in agreement with the assertion that OPB is used to further stabilize these targets. The current findings are clinically significant as OPB showed a notable enhancement in cognitive performance, accompanied by a reduction in neuroinflammatory cytokines and oxidative stress in the LPS-induced neurotoxicity model. As neuroinflammation and redox imbalance are key factors in the progression of Alzheimer’s disease, Parkinson’s disease, and other cognitive disorders, the modulation of IL-6, IL-1β, TNF-α, SOD, GSH, CAT, and MDA, along with the normalization of TGF-β, NF-κB, and IκB-α, suggests that OPB may serve as a promising therapeutic strategy. Additionally, in silico docking and dynamic simulations supported its mechanistic plausibility, demonstrating stable and favorable interactions with NF-κB and IκB-α, which are two crucial regulators of neuroinflammatory processes. Therefore, the in-silico results support the fact that OPB targets the NF-κB/IκB-α axis as a likely process to reduce neuroinflammation and consequent cognitive impairment induced by LPS (Fig 13).

Despite the results from both in vivo and in silico models, this study has certain limitations, such as the small number of experimental subjects and the duration of the study (7 days), which may not completely explore the therapeutic potential of OPB. This study mainly included behavioral and biochemical assessments and lacked downstream validation of pathway-specific markers using western blotting, transcriptomics, Iba-1 immunostaining, and immunohistochemistry. Therefore, a detailed analysis of the molecular processes associated with the therapeutic effects of OPB in managing LPS-induced neurotoxicity and associated learning and cognitive deficits is required. It is important to note that the previously evaluated NF-κB-targeted anti-inflammatory agents have produced inconsistent results. These discrepancies may stem from variations in the experimental models, dosing regimens, treatment timelines, and endpoint measurements. Specifically regarding OPB, there are limited comparative data in the context of neuroinflammation, which calls for careful interpretation of the findings. The current results offer strong preclinical justification, and it is crucial to conduct multi-model studies, evaluate chronic dosing, and initiate early phase clinical investigations to fully understand OPB’s potential of OPB for translation into clinical practice.

## Conclusion

The results of the in vivo analysis, strengthened by in silico methods, strongly support OPB as a potential therapeutic alternative for ameliorating neurotoxicity and associated memory and learning impairments by modulating neuroinflammation and oxidative stress. The significant improvements observed in cognitive performance in the murine model suggest that OPB has therapeutic properties for neuronal conditions. However, the important translational gaps remain unexplored, and the current results concur with short-term assessments only in male rats, and thus need to be validated for long-term effects and comorbidity-inclusive models to represent clinical scenarios. Gene-specific studies are required to confirm the involvement of these pathways. Thus, detailed assessments should be conducted to explore the molecular mechanisms associated with the therapeutic effects of OPB in countering the cognitive deficits caused by neurotoxicity. Future studies should incorporate multiple time-points to capture both acute and delayed responses, thereby strengthening the translational relevance of our findings.
